# Miniaturizing Nanotoxicity Assays in Daphnids

**DOI:** 10.3390/ani14142046

**Published:** 2024-07-12

**Authors:** Dimitrios Kakavas, Konstantinos Panagiotidis, Keith D. Rochfort, Konstantinos Grintzalis

**Affiliations:** School of Biotechnology, Dublin City University, D09 Y5NO Dublin, Ireland; dimitrios.kakavas2@mail.dcu.ie (D.K.); konstantinos.panagiotidis@dcu.ie (K.P.); keith.rochfort@dcu.ie (K.D.R.)

**Keywords:** *Daphnia magna*, nanoparticles, silver nano ink, toxicity, miniaturization, enzyme kinetics, mortality, feeding rate

## Abstract

**Simple Summary:**

Applications of nanoparticles as well as their use have increased over the past decades without sufficient research on their environmental impact. Focusing on *Daphnia magna*, a widely adopted aquatic organism employed to evaluate the adverse effects of pollutants, we investigated several aspects of the experimental design used in *D. magna* toxicity testing which could impact the observed effect of exposure to nanoparticles. Furthermore, we evaluated the feasibility of a miniaturized version of *D. magna* toxicity test as a potential candidate for nanoparticle toxicity assays. Results showed that the exposure vessel and its characteristics can affect the impact of nanoparticles and, therefore, skew the observed effects. Additionally, the miniaturized exposure showed that for physiology markers such as toxicity and feeding rate it can be a good alternative to the traditional setups, as it vastly reduces the amount of media and number of nanoparticles required as well as generated waste.

**Abstract:**

The rapid progress of the modern world has resulted in new materials and products created at an accelerating pace. As such, nanoparticles have widespread applications and often find their way into the aquatic ecosystem. In the case of freshwater ecosystems, one of the commonly used bioindicators species used for pollution assessment is *Daphnid magna*. The Organization for Economic Co-operation and Development (OECD), and other organizations such as the European Chemicals Agency (ECHA) and Environmental Protection Agency (EPA), have set guidelines for acute toxicity testing in daphnids that are severely lacking in terms of information on the characteristics of the exposure vessel when studying the adverse effects of nanoparticles (NPs). Understanding the toxicity mechanisms of nanomaterials is imperative given the scarcity of information on their adverse effects. Furthermore, miniaturization of nanotoxicity assays can reduce the number of daphnids used, as well as the cost and nanomaterial waste, and provide results even at the individual animal level with enhanced reproducibility of testing. In this study, the impact of the exposure vessel on the observed physiological changes of daphnids was investigated for a silver nano ink. Exposures in eleven commercially available vessels; nine made of plastic and two made of glass were compared for 24 h. The effect of surface to volume ratio of the exposure vessel and the animal number or “crowding” during exposure was investigated in the context of miniaturizing biomarker assays as alternatives to traditional experimental setups in *Daphnid magna*. Toxicity curves showed differences depending on the vessel used, while a novel feeding rate assay and the activity of key enzymes were assessed as physiology endpoints.

## 1. Introduction

Nanomaterials (NMs) present an emerging threat in the ecosystem as a result of their increased use over the last number of decades. Commercial use of NMs has increased exponentially since 1990 [1,2,3], with NMs present in many consumer products; therefore, increasing the possibility of accidental release into the environment. Industry and medical domains are the most prevalent in terms of NM applications [4,5,6,7]. In relation to their entry into the environment, the primary routes are through sewage effluents (industrial or municipal), insufficient waste treatment, handling of products containing NMs and accidental spills [8]. Material degradation and surface wear can also result in creating additional nanoparticles (NPs) that ultimately enter the environment. The unintentional generation of NPs is a matter of grave concern, since there is evidence of NP bioaccumulation and toxicity phenomena while there is little understanding of the factors that can affect NP bioavailability and toxicity mechanisms to aquatic life. With scarce information over the impact of engineered NMs on aquatic life, their release poses a novel environmental threat. Multiple characteristics of NPs influence their toxicity to aquatic life and these include their physicochemical properties related to their shape, size, surface charge and coating [9,10,11].

Metallic NPs in the form of nano inks are currently used in 3D printed electronics, a relatively new industrial domain that has gathered significant traction thanks to its potential to mass produce low-cost, highly customizable electronics [12]. Multiple studies have already reported the adverse effects of metallic and non-metallic NPs in bioindicator species; however, there is not sufficient information on the mechanistic aspect of toxicity for NPs in a solution [13,14,15]. Therefore, there is an urgent need to properly monitor these novel pollutants as well as expand the understanding of their toxicity mechanisms. Nanotoxicity assays conducted using aquatic organisms often utilize different experimental setups across laboratories [13,16,17,18,19,20,21]. In many cases, high volumes of NP stock suspensions are used, while the exposure vessel volume [22], surface-to-volume ratio [23] and animal number [24] have not been thoroughly assessed or their potential impact on acute toxicity testing. Other problems that arise are the generation of pollutants from toxicity testing, as well as the inconclusive and divergent results of nano-ecotoxicological studies [25]. In tandem with the great need to properly monitor and assess NPs as emergent pollutants, these two critical problems must be urgently addressed. Using bioindicator organisms such as *Daphnid magna* and *Danio rerio* for toxicity testing can provide critical insights into the toxicity of different NPs and presents several advantages over traditional approaches to pollution assessment [26,27,28]. Daphnids are key freshwater species in nanotoxicology and ecotoxicology as biological systems that can provide meaningful conclusions on the impact of pollutants [29]. Exposing bioindicator species to pollutants allows for a range of biomarkers such as immobilisation [30], growth [31], reproduction [32], feeding rate [33], survival [34] and more, to be utilized in order to evaluate the physiological impact of exposure, therefore enabling the detection pollutants at a very critical early stage [35]. The utilization of daphnids as bioindicator species to serve as sensitive indicators for environmental changes can enable the detection and identification of potential hazards before they have the capacity to cause serious adverse effects on aquatic life in freshwater ecosystems [35,36,37]. Their responses to pollutant exposure can provide valuable information on the toxicity levels of novel pollutants such as NPs, and provide critical insight on the underlying mechanisms of toxicity [38,39,40,41]. Additionally, the use of bioindicator species allows for the assessment of various adverse effects on their physiology, providing a comprehensive understanding of the potential risks associated with NP pollution [19,30,42,43].

In this study, silver nano ink was used to study the impact of exposure in a miniaturised toxicity assay setup as a potential alternative to traditional experimental setups of *D. magna*. Additionally, exposure vessels which differ from one another with respect to their characteristics such as volume, surface-to-volume ratio (which reflects the shallowness or depth of the exposure vessel) and the animal number (or crowding) were assessed in acute exposures. Using lethality as a surrogate measure of toxicity, a novel feeding assay and biochemical markers, the impact of nano inks on daphnids was assessed under different exposure conditions.

## 2. Materials and Methods

### 2.1. Culturing of Daphnids and Toxicity Exposures

Cultures of daphnids were maintained in glass beakers in aqueous media (final concentrations 0.29 g CaCl_2_.2H_2_O/L, 0.123 g MgSO_4_.7H_2_O/L, 0.065 g NaHCO_3_/L, 0.0058 g KCl/L, 2 μg Na_2_SeO_3_/L, pH 7.7) under a 16 h:8 h of light:dark photoperiod at 20 °C, at a density of 80 adults per 4 L of media. Media were renewed every 5 days and cultures were fed daily with an algal (*Chlamydomonas rheinhartii*) suspension and 15 mL of an organic seaweed extract (*Ascophylum nodosum*) only upon media renewal. For nano exposures, neonates (<24 h) were collected and cultured until they were 4 days old and fed daily with algae (60 million cells). On the fourth day, animals were relocated to different vessels for exposure and mortality was assessed following 24 h. The vessels used were a 6-well plate (6wp), a 12-well plate (12wp), a 24-well plate (24wp), a 48-well plate (48wp), a 96-well plate (96wp), a cuvette (C), a Petri dish 50 mL (P50), a Petri dish (100 mL), a centrifuge tube (f), a glass vessel 50 mL (G50), and a glass vessel 100 mL (G100). Toxicity curves were plotted using the four-parameter logistic (4PL) model, following the equation Span = Top − Bottom and Y = Bottom + (Top − Bottom)/(1 + 10^((LogIC_50_ − X) × HillSlope)), using the GraphPad software 10.2. The EC values were calculated and a concentration of 0.5 μL nano ink/L (derived from the data of the toxicity curves) was used as sub-lethal exposure concentration for the enzyme activity and feeding rate exposures of 24 h. Silver nanoparticle ink was purchased from Sigma-Aldrich to conduct the exposure experiments (Product No. 719048). The silver nanoparticle ink with this product code has been altered to wt 20%; however, when originally purchased the product was different with wt 50%. In this study, we selected to work with four-day-old daphnids based on other studies [23,35,37] focusing on older animals compared to neonates which would be more sensitive to pollutants. In addition, four-day-old daphnids provide more tissue for biochemical assays and are easier due to their size to work with, while they can also give critical information on their response to pollutants compared to an early adolescent life stage of the organism.

### 2.2. Sample Homogenization and Biochemical Assays

Four-day-old daphnids were exposed to nano ink (0.5 μL/L) for 24 h. Following exposure, 30 daphnids were combined and homogenized in 0.5 mL of phosphate buffer (pH 7.2) using an Eppendorf pestle. The homogenate was cleared by centrifugation (12,000× *g* for 10 min at 5 °C) and the clear supernatant was collected and immediately assayed for protein and enzyme activity [35,44]. The highly concentrated homogenate was further diluted as it is necessary to obtain unit values within a reliable linear range for each kinetic assay. Protein quantification was performed using a sensitive Bradford method [4,45] to normalise enzyme activity in units/μg protein.

Phosphatase activity was determined for the released *p*-nitrophenol utilizing *p*-nitrophenyl phosphate (*p*NPP) as substrate, in 100 mM citric acid pH 4.5 (for ACP) or 100 mM boric acid pH 9.8 (for ALP) [44]. The reaction volumes were set to 200 μL sample appropriately diluted in buffer and mixed with 50 μL 8 mM *p*NPP. The reaction was alkalized after 30 min with the addition of 50 μL 4 M NaOH, and the absorbance was measured at 405 nm and converted to *p*-nitrophenol from the corresponding standard curve. Similar to the aforementioned endpoint kinetics, the activities of β-galactosidase (BGAL) and lipase (LIP) were determined, by measuring the generation of nitrophenol resulting from the catalysis of *o*-nitrophenyl-β-galactoside and *p*-nitrophenyl butyrate, respectively, in 50 mM phosphate buffer pH 7.2.

The activity of γ-glutamyl transferase (peptidase) was quantified using L-leucine-4-nitroanilide as a substrate [46]. For the quantification, 200 μL of appropriately diluted sample in 50 mM phosphate buffer pH 7.2 was mixed with 50 μL of 8 mM substrate in 100% DMSO, and the release of the product *p*-nitroanilide was monitored continuously at 418 nm. The activity of GST was determined by monitoring the formation of a complex between reduced glutathione and 1-chloro-2,4-dinitrobenzene and measured photometrically at 340 nm [47]. The activity of LDH was quantified by measuring the decrease in absorbance at 340 nm resulting from the oxidation of NADH. The substrate employed was a 1:1 mixture of 40 mM pyruvate and 0.5 mM NADH. For the reaction, 200 μL of the sample appropriately diluted in phosphate buffer was mixed with 50 μL of 20 mM pyruvate and 0.25 mM NADH, and the oxidation of NADH to NAD^+^ was continuously monitored at 340 nm. Reduced thiols (RT) were quantified through their reaction with aldrithiol following the protocol of Grintzalis et al. [48].

### 2.3. Feeding and Imaging of Daphnids

The feeding rate was assessed as a phenotypic physiology endpoint. Typically, methods employ algae and long incubation periods; however, in our approach we used a novel method based on the tracking of ingested fluorescent microparticles (carboxylate-modified fluorescent latex beads, CAT number: L3030), which were were purchased from Sigma Aldrich (St. Louis, MO, USA) [49]. Daphnids were collected following a 24-h exposure to silver nano ink (0.5 μL/L) and incubated with 18 mL of fluorescent microparticles for 30 min to evaluate the feeding rate of daphnids. For the feeding assay, 4 replicates of 15 animals each were utilized to evaluate the impact of exposure on the feeding rate. Fluorescence from microparticles was measured at Ex/Em 560/590 nm using a TECAN plate reader (Männedorf, Switzerland). Fluorescence measurements from the media were converted to the amount of ingested microplastics using a standard curve and expressed as feeding rate per animal.

### 2.4. Statistical Analysis

Results were presented as mean ± standard deviation (SD) and were analysed and plotted with GraphPad Prism software 10.2. Statistically significant differences were identified using one-way ANOVA and a Student’s *t*-test with a Welch’s correction over the unexposed control condition with a *p* value of 0.05.

## 3. Results

### 3.1. Exposure to Silver Nano Ink in Different Vessels and Volumes Impacts Mortality

**Figure 1:**
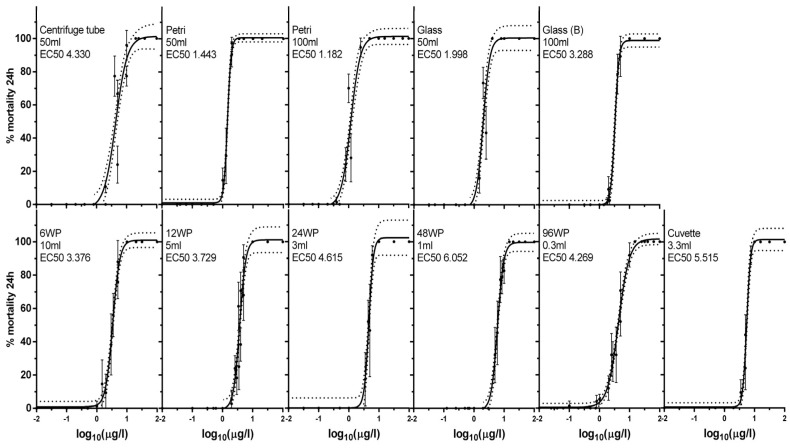
Acute toxicity curve for AgNP nano ink in 11 exposure vessels. EC_50_ values are in μL/L. Fifteen 4−day−old daphnids per replicate of each concentration tested were exposed to AgNPs nano ink for 24 h. Data represent average ± SD (N = 5). Using the four-parameter logistic (4PL) model, the EC_50_ values were calculated.

Acute exposure of daphnids to the silver nano ink for 24 h was assessed in different volumes and vessels with full toxicity curves (Figure 1) and the EC_50_, EC_1_ and Hill slope were calculated (Table 1). Full toxicity curves were generated with a minimum of three independent experiments per exposure vessel conducted, to reduce any possible difference between batches of daphnids in the observed mortality. The lowest EC_50_ value (0.221 μL/L of the nano ink stock) was observed in the P100 (Petri 100 mL) exposure condition while the highest (5.515 μL/L of nano ink stock) was observed in the cuvette condition (Table 1).

**Figure 2:**
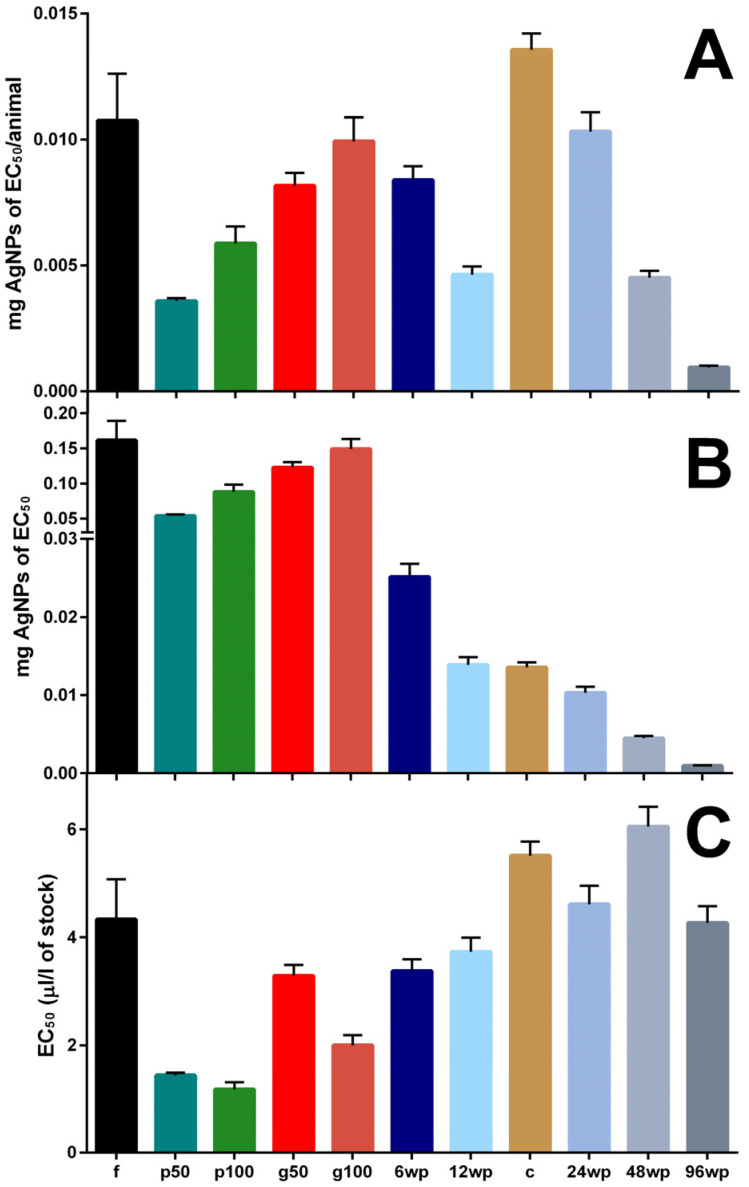
EC_50_ values of all 11 vessels tested were calculated with the four-parameter logistic (4PL) model. (**A**,**B**) show the mg of AgNPs calculated that are equivalent to the EC_50_ for each vessel tested, per replicate and per animal, respectively. (**C**) depicts the amount of silver nano ink in μL/L equivalent to the EC_50_ for each vessel tested. The error bars in the figure depict the range of data, with each bar indicating the minimum and maximum values (Table 1).

Hill Slope values also deviated across the tested conditions, ranging from 2.248 (centrifuge tube) to 7.321 (Petri 50 mL), showing great differences in steepness and the range of observed toxicity to the exposed daphnid. There were no visible signs of precipitation of AgNPs observed during the exposures. The characteristics of the vessel significantly impacted the observed effect on mortality during exposure; however, mortality does not follow any specific pattern among the tested vessels (Figure 2). For the following exposures and assessment of other biomarkers, 0.5 μL/L was selected as the exposure concentration due to being close to the equivalent EC_1_ values for the f (centrifuge tube), 96wp (96-well plate) and the g50 (glass 50 mL) exposure conditions.

The top Figure 2 (mg AgNPs/daphnid) shows that tall vessels require more NPs per daphnid during exposure in order to achieve the same % of lethality in a tested group. The NP amount per daphnid that is calculated as equivalent to the EC_50_ for each condition tested shows great variation across all conditions. The miniaturisation approach for the experimental setup results in a significant decrease in the NP amount that is equivalent to the EC_50_, potentially presenting a more accurate and realistic depiction of the AgNPs exposure effect when focusing on single individuals of *D. magna* compared to larger test vessels. The 96wp required the smallest amount of AgNPs that is equivalent to the EC_50_ concentration during exposures. The middle Figure 2 (mg AgNPs equivalent to EC_50_) shows that the AgNP amount of EC_50_ in each condition follows a decrease that mostly correlates to the decrease in the test vessel’s volume. These observations also confirm that smaller vessels require fewer AgNPs to produce the same effect of exposure. The miniaturisation approach of using small volume vessels shows a trend of decrease for the mg of AgNPs equivalent to the EC_50_ of each condition that correlates and matches to the decrease in the exposure volume in all exposure vessels. In the bottom Figure 2, we can observe the differences in amount of silver nano ink stock required in each test condition. Furthermore, in the large exposure vessels with a high S:V (petri 50 mL, petri 100 mL) the stock amount required is also significantly less when compared to other large vessels. The only exception is a miniaturised exposure vessel, the 96wp which, in spite of having a high S:V, requires more silver nano ink stock for the EC_50_ concentration when compared to vessels with a smaller S:V.

**Figure 3:**
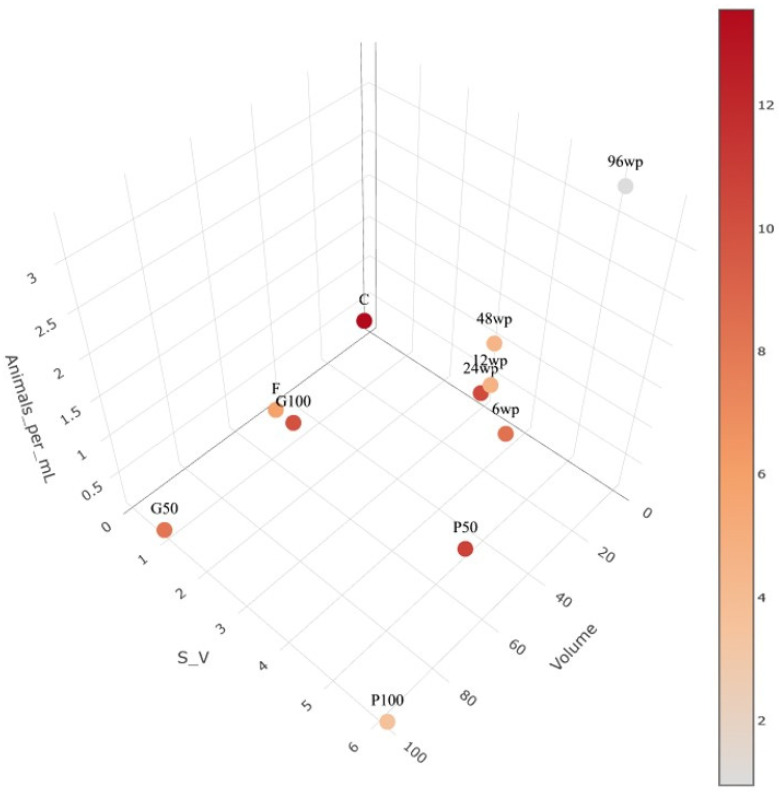
The impact of vessel on mortality. Points in the figure correlate to each exposure vessel with axes used to plot the characteristics of the vessel. Colour intensity represents the amount of AgNP nano ink (mg/daphnid in the exposure vessel) for the EC_50_. A supplementary version of this image with free rotation is provided.

The initial assessment of the different exposure vessel mortality during AgNP nano ink exposure revealed that the higher-volume vessel does not always require greater amounts of AgNPs compared to a miniaturised approach, as seen by the C (cuvette) condition (Figure 3). However, the deviation in EC_50_ values in tested vessels clearly shows an influence of the different vessel characteristics on the toxicity of AgNPs. During the exposure period, no mortality was observed in control exposures that had no AgNPs added. These conditions were added to ensure the good quality of the brood during exposure. The lowest EC_1_ value calculated was in the petri 50 mL exposure vessel (0.221 μL/L of silver nano ink stock), and the highest was calculated in the cuvette condition (2.807 μL/L of silver nano ink stock). The differences in vessel characteristics for S:V, animals, animals/mL and volume for the 11 conditions tested can be observed in Table 1.

### 3.2. Physiology Responses following Exposure to the Silver Nano Ink

**Figure 4:**
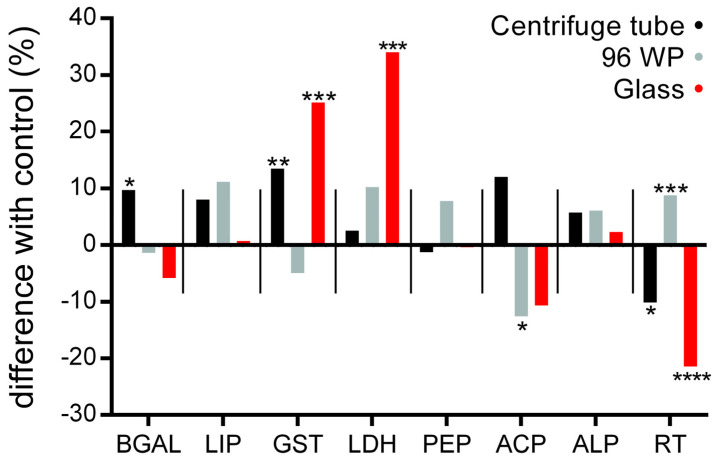
Percentage of difference in enzyme activity due to exposure to silver nano ink (0.5 μL/L, 24 h) in different exposure vessels (each vessel was compared to their corresponding control). Data represent average ± SD (N = 4) replicates for each condition. Statistically significant changes identified by one-way ANOVA between control and exposed conditions with *p*-values of *p* ≤ 0.05 (*), *p* ≤ 0.01 (**), *p* ≤ 0.001 (***) and *p* ≤ 0.0001 (****). All data values are provided in Supplementary Tables. Abbreviations: BGAL: β−galactosidase, LIP: lipase, GST: glutathione S−transferase, LDH: lactate dehydrogenase, PEP: peptidase, ACP: acidic phosphatase, ALP: alkaline phosphatase, RT: reduced thiols.

Daphnids were exposed to 0.5 μL/L of silver nano ink and responses in key enzyme activity were recorded (see Supplementary Tables). Comparisons between control and nano exposed but also across the exposed conditions reveal statistically significant differences. Biochemical responses (Figure 4) showed differences between the centrifuge tube, 96-well plate and glass vessel on the comparison of observed enzyme activity levels but also when exposure vessels were compared to their respective controls. The statistically significant differences in enzyme activity across the eight tested enzymes do not follow a specific trend and change both in the enzymes and observed intensity across the tested conditions. The greatest difference in the enzyme activity was observed in the activity of LDH in the glass exposure condition (+34%) and the lowest for the reduced thiols (−22%). The miniaturised approach in the 96wp shows a significant decrease in ACP activity (−22%) and an increase in reduced thiols (+9%). Furthermore, the centrifuge tube exposure condition shows a significant increase in GST activity (+13.5%) as well as in the activity of BGAL (+10%).

**Figure 5:**
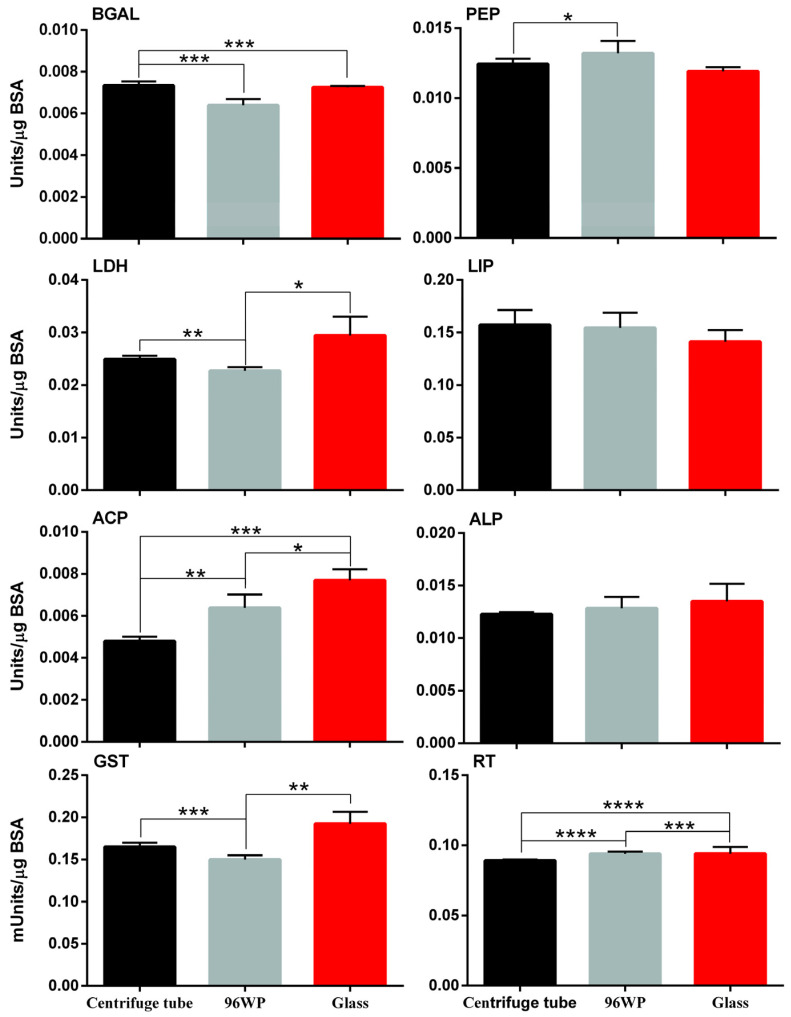
The impact of miniaturization and vessel material on controls. Comparisons of enzyme activities between the unexposed controls of a centrifuge tube, glass and 96-well plate exposure vessels. Data represent average ± SD (N = 4) replicates for each condition. Statistically significant changes identified by one-way ANOVA between control and exposed conditions with *p*-values of *p* ≤ 0.05 (*), *p* ≤ 0.01 (**), *p* ≤ 0.001 (***) and *p* ≤ 0.0001 (****). All data values are provided in Supplementary Tables. Abbreviations: BGAL: β-galactosidase, LIP: lipase, GST: glutathione S-transferase, LDH: lactate dehydrogenase, PEP: peptidase, ACP: acidic phosphatase, ALP: alkaline phosphatase, RT: reduced thiols.

Statistically significant differences in the eight enzymes and compounds tested show a different profile of exposure response to the AgNPs in exposed individuals. A different toxicity mechanism of AgNPs in the 96wp condition could explain the difference in GST and reduced thiols when comparing the 96wp with the centrifuge tube or the glass exposure conditions (Figure 5). Additionally, in the glass exposure condition GST and LDH activity were significantly impacted by the exposure whereas in the centrifuge tube exposure condition, GST activity is impacted less and LDH significantly less when compared to the equivalent enzymes in the glass exposure condition. According to our results, GST activity and reduced thiols in both the centrifuge tube and the glass greatly deviated from their corresponding values in the 96wp exposed conditions.

**Figure 6:**
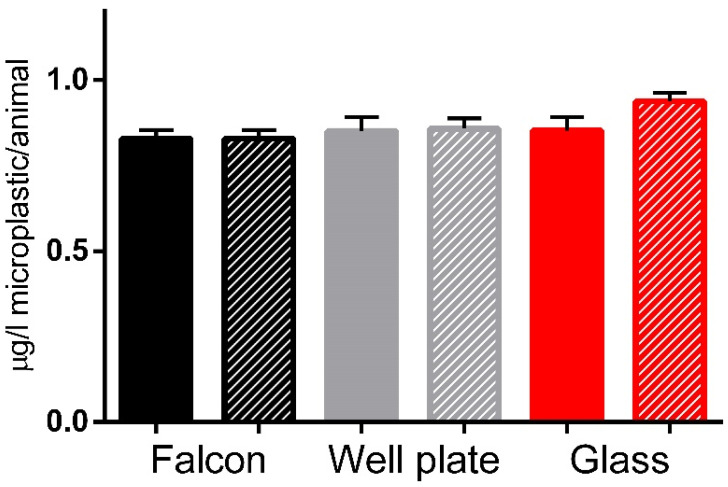
The impact of silver nano ink (indicated by striped bars, exposed at 0.5 μL/L for 24 h) compared to each vessel group’s control (non-striped bars) on the feeding rate of daphnids under different exposure vessels. Data represent the mean ± standard deviation (N = 4). No statistically significant differences using a Student’s *t*-test were found across the comparisons of exposure to control condition in the tested vessel groups. No statistically significant differences using one-way ANOVA were found in the comparisons among the control (solid) or among the exposed (striped) groups.

To provide a more holistic image on the effect of the exposure vessel on the physiology of daphnids, the enzyme activity of eight different enzymes was tested in the absence of silver nano ink (Figure 6). We observed statistically significant different enzyme amounts in six out of eight tested enzymes and compounds, with ALP and LIP being the two that displayed none. These observations confirm that the impact of the exposure vessel on the physiology of daphnids could in part explain the differences observed in other tested biomarkers.

However, the feeding rate showed no statistically significant differences between the tested conditions. These findings suggest the absence of impact on the daphnid feeding rate in the sub-lethal concentrations that were tested and could indicate that a miniaturized approach in the feeding rate assessment is feasible.

## 4. Discussion

In this study, the effects of different exposure vessels on the toxicity of AgNPs to *D. magna* were investigated on several biomarkers. Additionally, we investigated a miniaturized approach as a better alternative to traditional *D. magna* toxicity testing experimental setups, as well as studied how miniaturization can affect the observed impact in certain biomarkers of daphnid physiology [50,51]. The dynamic interactions between the exposure vessel and pollutants may affect the physicochemical properties of NPs and, therefore, their impact on exposure to daphnids. This also highlights the importance of the standardization of exposure vessels and protocols used across laboratories for *D. magna* toxicity testing. Our study hypothesizes that as NP solutions are colloidal in nature, when exposure vessels with different characteristics are used for pollutant assessment NPs can potentially interact with the vessel itself and the exposed daphnid in distinct ways. Consequently, certain vessel characteristics could influence the observed impact of NP exposure to bioindicator species used to study pollution such as *D. magna* [24]. There is a clear gap in knowledge concerning the influence of the exposure vessels on the observed impact of exposure on biomarkers of daphnid physiology, which are used to assess responses to pollutants such as mortality, feeding rate, etc. To the best of our knowledge, this is the only study that focuses on the impact of exposure vessels to observed toxicity and other biomarkers of daphnid physiology except for Gkrintzalis et al. [24], in which the researchers used a wide range of exposure vessels to evaluate the impacts of systematically varying the total media volume, the surface-to-volume ratio, and the animal density for the acute toxicity testing of cadmium. However, in said study, only mortality was used as a marker to evaluate the effect of different vessel characteristics. In this study, 11 vessels were assessed, selected with the criteria of being (1) commercially available, (2) used in ecotoxicological studies with daphnids, and (3) good candidates to test the viability of miniaturising acute exposures [52,53]. The vessel characteristics studied were the volume, number of animals in each vessel (crowding), S:V and vessel material (glass/plastic) on their impact to the observed effect of AgNP nano ink exposure to mortality, enzyme activity and feeding rate. According to our results (Figure 3), the higher to S:V, the observed mortality is lower but not in all vessels. This could be attributed to the fact that a NP solution is colloidal and therefore in vessels with capacity for large volumes (e.g., 50 mL) and that are tall, NPs will sink over time to the bottom of the vessel. However, the *D. magna* exposure to AgNPs results in an observable effect of exposure, since part of the toxicity effect of AgNP exposure is attributed to the leeching of Ag+ ions in the surrounding media [54]. The vessel volume alone is not a defining factor for the observed mortality in vessels but vessels with more volume generally resulted in higher observed toxicity to exposed *D. magna*, which can be seen when comparing the following groups in the context of their different volumes in Figure 3: p50/p100/96wp, g50/g100/96wp or instead of 96wp p50/p100/c, etc. Furthermore, the range of AgNPs concentration for the toxicity curve of each condition which can be correlated to the Hill Slope (Table 1) shows that the toxicity levels of AgNPs are impacted by the difference in the tested vessel characteristics, but do not follow a specific trend. The impact of animals per mL, or crowding, was also studied and according to mortality data (Figure 3), we cannot reach any clear conclusions regarding the impact of animal crowding.

According to our previous study [23], S:V impacts the toxicity of AgNPs. For example, the Petri dish as a shallow vessel, when compared to the centrifuge tube allows for easier access to AgNPs, possibly due to the large S:V, where the daphnid can only swim close to the bottom of the vessel while AgNPs will sink over the time of the acute exposure. This hypothesis is supported by the findings in Figure 2A,B where the amount of AgNPs in the centrifuge tube is equivalent to the EC_50_ for a single replicate and per animal is less when compared to the Petri dish. Additionally, the same observation can be made when comparing the AgNP amount of the AgNP silver nano ink concentration equivalent to EC_50_/animal for the 24wp and the cuvette, or the miniaturised version of 96wp when compared to any other exposure vessel (Figure 2A). The hypothesis we posit for this observation is the same as in our previous study which focused only on the S:V [23], and is that the restricted vertical movement of daphnids inside the exposure vessel, in tandem with the fact that the exposure media is a colloid solution is the reason for the increased impact of AgNPs on daphnids exposed in vessels with higher values of S:V, or more specifically, with a geometry that will restrict the daphnid to swim close to the bottom of the vessel during the exposure and possibly increase the availability of AgNPs. The impact of the vessel material can be observed when comparing the mortality in the f and g50 exposure conditions, where exposure in g50 seems to result in increased daphnid mortality. A possible explanation for this observation is that the AgNPs do not interact in the same way with glass and plastic, and this might result in different amounts of NPs being available in the media for the exposed daphnid, resulting in a different observed impact. However, there is very limited information on the adsorption capacity of glass surfaces and plastic surfaces in regard to AgNPs [55], and more research is required in order to fully understand the interactions between AgNPs and glass or plastic exposure vessels. Regarding the vessel impact on observed mortality, Figure 3 underscores a significant problem with the use of various vessels across studies: even with remarkably similar EC_50_ values, the true quantity of AgNPs per exposed individual can exhibit considerable variation. Therefore, a range of outcomes in terms of the observed effect of exposure can be expected for other, more sensitive biomarkers of daphnid physiology such as enzyme activity, survival assays or omics analysis. Another crucial aspect that needs to be considered is the geometry of exposure vessels which can inflict additional stress on the exposed daphnid as our results in Figure 5 highlight. The observed effect on the studied biomarkers shows that the choice of exposure vessels used in laboratory studies can result in significantly varying distributions of AgNPs to the exposed daphnid and therefore observed effect of exposure among different studies, even when the concentrations of the initial NP stock are similar.

After assessing the impact of various vessels on the observed mortality, three vessels were identified for additional investigation: f, g50 and 96wp. The selection was made based on the differences in vessel material and with the aim to further investigate the feasibility of miniaturization. The feeding rate was not impacted by the different exposure setups as it showed no differences between them. This absence of observed differences is a good indicator that a miniaturised experimental setup for *D. magna* exposures will not affect this commonly used biomarker in environmental studies. However, this is not the case for more sensitive biomarkers such as enzyme activity, which clearly show a distinctively different response in many key enzymes of *D. magna* physiology and therefore invalidate any comparison of such results across studies unless the same exposure vessels were used among them. According to [25], biochemical assays are a viable tool to examine on a more precise level the effect of pollutant exposures. Regarding the results of the enzyme activity assays (Figure 5), it is noteworthy that all control conditions exhibited statistically significant differences in the activity levels of six out of eight enzymes tested. The most notable variations were observed in the comparisons between the centrifuge tube and 96wp, with statistically significant differences detected in the activity levels of four enzymes (BGAL, PEP, LDH, ACP, GST) and reduced thiols. Comparison of the glass and the centrifuge tube control conditions displayed the fewest differences, with three out of eight enzymes having statistically significant differences, which we hypothesize is because the centrifuge tube and glass have the same exposure vessel volume (Figure 3). The fact that the controls of each tested vessel displayed a different pattern of statistically significant differences when compared to their respective exposed condition shows that in the context of studying the effect of AgNP exposure to very sensitive markers of daphnid physiology the vessel used for the exposures cannot be changed in favour of a miniaturized approach as this can result in inflicting additional stress or differences on a biochemical level for the exposed daphnid (Figure 3), skewing the observed. In the context of less sensitive markers such as mortality and the feeding rate, which in our study showed no statistically significant differences, the miniaturisation setup is a good potential alternative to traditional *D. magna* exposure setups. Furthermore, it may be actually a more accurate alternative for pollution assessment specifically for NPs, which due to their unique features require an alternative approach that is not affected by the S:V of the vessel or other vessel characteristics. Regarding the differences between the centrifuge tube and glass exposure vessels, the different factors between these two exposure vessels are the S:V and the vessel material. According to our previous study [23], we know that the difference in S:V can result in differences in observed toxicity as well as the activity of the eight enzymes tested. However, the S:V of the centrifuge tube and glass exposure vessels does not differentiate much in order to be the only responsible factor for any differences observed. Therefore, the vessel material may also be responsible for the deviation in enzyme activity. To the best of our knowledge, there is no available information that covers how different vessel material can influence NP toxicity, although there has been some research [56] which suggests that the test vessel’s parameters could impact the observed effect but this is focused mainly on the impact of surface stabilizers or dispersants.

In both centrifuge tube and glass exposure vessels, we observed either an increase in the case of GST activity or a decrease in the case of reduced thiols whereas the opposite is true for the 96wp. Since AgNPs are known to cause ROS and DNA damage to living organisms, the deviation in the observed enzyme activity values for GST and RT between centrifuge tube/glass and 96wp we hypothesize is observed because in the 96wp daphnid are more encumbered by the exposure to NPs in terms of oxidative stress and possibly hinting at a different toxicity mechanism [57,58]. Although the differences in the control values of enzyme activity indicate additional stress may be caused to daphnids during exposure due to the vessel characteristics, the miniaturized approach may be a more precise alternative to standard testing in daphnids regarding NP pollutants. Due to the geometry of the vessel, daphnids are limited in terms of their movements very close to NPs in the exposure media. Therefore, more realistic results can be obtained in terms of the true impact of AgNP exposure and how dangerous an NP pollutant is, due to the fact that the amount of NPs required to achieve these results is considerably less when compared to larger volume vessels (Figure 2). Additional research is required to fully evaluate the feasibility of a miniaturized approach of daphnid toxicity testing for more sensitive markers of daphnid physiology.

In conclusion, our study highlights the critical influence of exposure vessel characteristics on the observed toxicity of AgNPs to *D. magna*. These findings underscore the importance of standardizing exposure vessel parameters in ecotoxicological studies to ensure the reliability and comparability of toxicity assessment data for NP pollutants across studies. Our study also shows that certain biomarkers can be reliably used for NP pollution assessment in a miniaturised approach which potentially produces more accurate results compared to traditional experimental setups. This new approach vastly reduces the required amount of NPs for toxicity testing and shows that the required NP amount to observe adverse effects after exposure is significantly less compared to when using larger exposure vessels. Moving forward, further research is necessary to further explore the underlying mechanisms driving the observed correlations of toxicity, vessels characteristics and observed effect, and to develop comprehensive guidelines for the selection and use of exposure vessels in the toxicity assessment of NP pollutants. By addressing these considerations, we can improve the accuracy and relevance of NP toxicity assessments, ultimately contributing to more effective environmental risk assessment and management strategies.

## 5. Conclusions

The presented data highlight the absence of information relating to the impact of the characteristics of the exposure vessel on the observed effect of NP, and, therefore, the urgency for a standardized testing for the assessment of NP pollutants. Additional research is required to determine how much impact each individual test vessel parameter actually has, as well as for the possibility of using a miniaturized exposure setup in order to reduce generated pollutants during testing. This new miniaturized approach to the NP pollution assessment may also result in a more holistic understanding of the toxicity mechanisms of NPs without compromising the reproducibility of tests.

## Figures and Tables

**Table 1:** EC values (in μL/L) for silver nano ink acute exposures.

Vessel	6-Well Plate	12-Well Plate	24-Well Plate	48-Well Plate	96-Well Plate	Cuvette	Petri Dish (50 mL)	Petri Dish (100 mL)	Centrifuge Tube	Glass Vessel (100 mL)	Glass Vessel (50 mL)
Abbreviation	6wp	12wp	24wp	48wp	96wp	C	P50	P100	f	G100	G50
Volume (mL)	10	5	3	1	0.3	3.3	50	100	50	100	50
Animals	3	3	1	1	1	1	15	15	15	15	15
Animals/mL	0.3	0.6	0.33	1	3.33	0.3	0.3	0.15	0.3	0.15	0.3
S:V	3.98	3.32	3.03	3.14	5.13	0.03	5.09	6.16	0.49	0.92	0.96
EC_1_	0.974	1.264	2.060	2.042	0.629	2.807	0.77	0.221	0.561	0.615	1.605
EC_50_	3.376	3.729	4.615	6.052	4.269	5.515	1.443	1.182	4.330	1.998	3.288
(min–max)	(3.17–3.60)	(3.43–4.00)	(4.3–4.96)	(5.71–6.42)	(3.98–4.58)	(5.27–5.78)	(1.36–1.49)	(1.06–1.32)	(3.69–5.08)	(1.82–2.19)	(3.1–3.49)
Hill slope	3.697	4.246	5.696	4.230	2.400	6.804	7.321	2.744	2.248	3.898	6.407

## Data Availability

All raw data from this study will be provided upon request. Additionally, data from Figure 2 and Figure 3 are provided in Supplementary Materials in table format.

## Supplementary Materials

The following supporting information can be downloaded at: https://www.mdpi.com/article/10.3390/ani14142046/s1, Supplementary Table S1: The acute effect of miniaturization on multiple enzymatic markers of daphnids upon exposure to silver nano ink; Supplementary Table S2: The acute effect of miniaturization on multiple enzymatic markers of daphnids. Supplementary File S1 with rotation for all expressions of nano ink.
