# Supplementary material for: Miniaturizing Nanotoxicity Assays in Daphnids
Source: Animals (Basel). 2024 Jul 12;14(14):2046. doi: 10.3390/ani14142046 (PMC11274355; doi:10.3390/ani14142046)
Supplement: Supplementary file 1 [file animals-14-02046-s001.zip › Supplementary.pdf]

Supplementary Table S1. The acute effect of miniaturization on multiple enzymatic markers of daphnids after exposure to silver nano ink. Data represents average±SD (N=4) replicates for each condition in units/mg BSA. Statistically significant changes identified by Student's *t* test between control and exposed (0.5 µl/l of silver nano-ink for 24 hours) condition (*). Statistically significant changes identified by one-way ANOVA between control and exposed conditions with *p*-values of $p \leq 0.05$ (*), $p \leq 0.01$ (**), $p \leq 0.001$ (***) and $p \leq 0.0001$ (****). Abbreviations: BGAL: β-galactosidase, LIP: lipase, GST: glutathione-S-transferase, LDH: lactate dehydrogenase, PEP: peptidase, ACP: acidic phosphatase, ALP: alkaline phosphatase, RT: reduced thiols.

Enzyme	Falcon		96 well plate		Glass vessel	
	Control	AgNPs	Control	AgNPs	Control	AgNPs
BGAL	6.4±0.3	7.02±0.27 **(+10%)***	7.25±0.06	7.15±0.28	7.34±0.2	6.91±0.45
LIP	154.47±14.61	166.81±8.94	141.35±10.84	157.11±3.45	157.2±14.28	158.32±10.23
GST	0.15±0.01	0.17±0.01 **(+14%)****	0.19±0.01	0.18±0.01	0.16±0	0.21±0.01 **(+25%)****
LDH	22.71±0.72	23.28±1.84	29.48±3.56	32.49±3.38	24.94±0.66	33.42±2.1 **(+34%)****
PEP	13.21±0.88	13.04±1.49	11.91±0.29	12.84±1.16	12.43±0.39	12.38±0.62
ACP	4.78±0.22	5.36±0.62	6.38±0.64	5.58±0.48 **(-13%)***	7.7±0.51	6.88±0.37
ALP	12.27±0.21	12.98±1.2	12.85±1.1	13.63±1.03	13.51±1.67	13.82±1.16
RT	93.81±1.64	84.29±2.43 **(-10%)***	93.94±4.91	102.14±4.04 **(+9%)****	89.2±0.73	70.06±0.57 **(-21%)******

**Supplementary Table S2. The acute effect of miniaturization on multiple enzymatic markers of daphnids.** Data represents average±SD (N=4) replicates for each condition in units/mg BSA. Statistically significant by one-way ANOVA (*), ($), (#) denotes between control conditions for comparisons between falcon and glass (*), 96wp and falcon tubes ($), and glass and 96wp (#).Statistically significant changes identified by one-way ANOVA between control and exposed conditions with *p*-values of $p \leq 0.05$ (*), $p \leq 0.01$ (**), $p \leq 0.001$ (***) and $p \leq 0.0001$ (****).			
**Enzyme**	**Falcon**	**96 well plate**	**Glass vessel**
BGAL	**6.4±0.3 (-15%)****	**7.25±0.06 (-13%)$^{$$}$**	7.34±0.2
LIP	154.47±14.61	141.35±10.84	157.2±14.28
GST	**0.15±0.01 (-10%)******	0.19±0.01	**0.16±0 (+17%)$^{\#\#}$**
LDH	22.71±0.72	**29.48±3.56 (-30%)$^{$$}$**	24.94±0.66
PEP	13.21±0.88	11.91±0.29	12.43±0.39
ACP	**4.78±0.22 (-61%)*****	6.38±0.64 **(+33%)****	7.7±0.51 **(+17%)$^{\#\#}$**
ALP	**12.27±0.21 (+21%)****	12.85±1.1	**13.51±1.67 (+22%)$^{\#\#\#}$**
RT	93.81±1.64	93.94±4.91	89.2±0.73
